# The role of carotid stenosis ultrasound scale in the prediction of ischemic stroke

**DOI:** 10.1007/s10072-019-04204-8

**Published:** 2020-01-03

**Authors:** Yi Tang, Ming-yu Wang, Tao-tao Wu, Jian-yu Zhang, Ru Yang, Bo Zhang, Ying Shi, Pin Meng, Niu Ji, Yongan Sun, Ying-da Xu, Bing-chao Xu, Xin-yu Zhou, Xiao-bing He, Guang-hui Zhang, Xiao-qin Niu, Zai-po Li, Bei Wang, Bei Xu, Zeng-lin Cai, Yong-jin Zhang, Ming-li He

**Affiliations:** 1 Department of Neurology, The Lianyungang Hospital Affiliated to Xuzhou Medical University, 182 Tongguan North Road, Lianyungang, 222002 Jiangsu China; 2 The Vascular Ultrasound Department, The Lianyungang Hospital Affiliated to Xuzhou Medical University, Lianyungang, Jiangsu China; 3 The Radiology Department, The Lianyungang Hospital Affiliated to Xuzhou Medical University, Lianyungang, Jiangsu China

**Keywords:** Brain ischemic, Carotid stenosis, Ultrasound, Neuroimaging, Cerebral blood flow

## Abstract

**Introduction:**

To improve the accuracy of ultrasound techniques for the assessment of carotid stenosis, we designed a novel carotid artery stenosis ultrasound scale (CASUS), and evaluated its accuracy, reliability, and its value in predicting the occurrence of cardiovascular and cerebrovascular diseases in a prospective study.

**Methods:**

A total of 750 patients with first-time ischemic stroke and hospitalized within 24 h were enrolled in the study. Using color Doppler ultrasound (CDUS), the degree of stenosis and blood flow (BF) in bilateral internal carotid arteries (ICA) and the V1-V3 segment of vertebral arteries (VA) was assessed. Cubic simulation curves for BF and global blood flow (GBF) over the stenosis score (SS), total stenosis score (TSS), and radiological imaging- total stenosis score (RI-TSS) were fitted and compared. The receiver operating characteristic (ROC) curves using TSS, RI-TSS, or GBF to predict various ischemic stroke endpoints were also analyzed and compared.

**Results:**

There was a linear relationship between SS and BF both ICA and VA (R2 were 0.734 and 0.783, respectively, both *P* < 0.05). Both TSS and RI-TSS with GBF showed an inverse “S” curve relationship (R2 was 0.839 and 0.843, all *P* < 0.05). The AUC values of TSS-based and RI-TSS-based predictions of each endpoint were all greater than 0.7 (all *P* < 0.05), but the differences of the AUC values between TSS, RI-TSS, and GBF were not statistically significant (all *P* > 0.05).

**Conclusions:**

The novel CASUS can better reflect the level of cerebral reperfusion in patients with ischemic stroke and can better predict the occurrence of cardiovascular and cerebrovascular diseases.

**Electronic supplementary material:**

The online version of this article (10.1007/s10072-019-04204-8) contains supplementary material, which is available to authorized users.

## Introduction

There is a general consensus that cerebral vascular stenosis leads to ischemic stroke since 1980s [1–4]. Previous reports had shown asymptomatic patients with carotid stenosis have an absolute stroke risk of over 3% in 1 year and a relative stroke risk of over 50% [5]. The results raise attention to the importance of kind, location, extent, or severity of the cerebral artery disease in the process of ischemic stroke. The new multi-mode molecular imaging technology has provided adequate technical support; however, it does not have a significant advantage on economical benefits, non-trauma, or non-ionizing radiation as compared with ultrasound [6]. As ultrasound is a rapid, easy, and sensitive method to evaluate carotid stenosis, it can be applied for large-scale screening and postoperative follow-up. Currently, the degree of carotid stenosis is measured based on the diameter of the carotid artery relative to the carotid bulb as shown by conventional angiography, the distal diameter of the carotid bulb [7–9] and the percentage lumen diameter reduction of the common carotid artery [10]. It is roughly classified into mild stenosis (with < 50% narrowing), moderate stenosis (50–69%), severe stenosis (70–99%), and occlusion [7]. This method categorizes carotid stenosis without further refinements; therefore, we hypothesize that the accuracy of carotid stenosis severity quantification could be improved by refining the NASCET method. A novel carotid artery stenosis ultrasound scale (CASUS) has been tested in the study and evaluated its accuracy and reliability as compared to computed tomography angiography (CTA).

## Materials and methods

### Study subjects

For this study, the inclusion criteria are as follows: (1) The patient must have a first-time, confirmed acute stroke and be within 24 h of the stroke onset; (2) The patient must have been confirmed to have at least one segment of stenosis in the internal carotid artery (ICA) or the V1-V3 segment of the vertebral artery (VA); (3) The patient or his/her proxy must have declined any carotid revascularization procedure such as stent placement and carotid endarterectomy; and (4) The patient or his/her proxy must have signed an informed consent and voluntarily participate in the study. The exclusion criteria are (1) severely impaired consciousness defined as NIHSS 1a consciousness score > 1, (2) modified Rankin scale (mRS) score > 0 prior to the stroke, (3) past history of carotid revascularization, (4) cardiogenic and cryptogenic stroke, (5) critical cerebral infarction, and (6) severe systemic disease and life expectancy < 6 months.

This study enrolled 846 patients with acute ischemic stroke who were hospitalized in our department from January 2016 to December 2016. Ninety-six patients were excluded according to the inclusion and exclusion criteria of this study. The final 750 patients were included in the analysis and follow-up (Supp. Fig. I Study protocols, data definitions, stenosis degree, and blood flow assessments and treatments, see the supplementary materials).

### CASUS scoring criteria and method

On the basis of the anatomy and basic hemodynamics of the carotid arteries [11], we speculated that as the degree of carotid stenosis increases, its BF linearly decreases. We hence proposed the following novel CASUS scoring system: mild stenosis (< 50%) = 1, moderate stenosis (50–69%) = 2, severe stenosis (70–99%) = 3, and occlusion (100%) = 4. If there is more than one stenosis in one blood vessel, the narrowest part of the blood vessel is used as the stenosis score for that blood vessel. The TSS of a patient is equal to the sum of the stenosis scores of the bilateral common carotid arteries, extracranial segments of bilateral ICAs, and V1–V3 segments of bilateral VAs. The total stenosis score (TSS) described is the CASUS score, and the total stenosis score indicated by CTA or MRA imaging examination using the CASUS criteria is expressed as RI-TSS (Angiographic assessments, see the supplementary materials). The RI-TSS was calculated in accordance with the CASUS criteria described above on the CTA or MRA images.

### Follow-up and endpoint definition

The study end points were assessed by qualified investigators at the time of admission and on the 30th, 90th, and 180th days from stroke onset. The end points are defined as follows: (I) primary end point, the time to recurrence of symptomatic stroke (ischemic or hemorrhagic, and fatal or nonfatal) and (II) secondary end points, the time of occurrence of the following events: (a) composite cardiovascular events (recurrent stroke, cardiac death, and non-fatal myocardial infarction) and (b) all-cause death [12].

### Statistical analyses

Statistical analyses were performed using SPSS20.0. The measurement data were expressed as mean ± standard deviation (±s), and the count data were expressed as percentages (%). The descriptive statistics method was used to calculate the corresponding relationship between the BF and SS of carotid stenosis of varying degrees. BF reduction was calculated as the difference between the mean BF with no stenosis and the mean BF with stenosis. SS was calculated as the quotient of the BF reduction normalized by the reference BF reduction (defined as the BF reduction of mild stenosis). Because BF cannot be detected in the case of occlusion, its BF was taken as 0 and SS as 4. The sample mean and standard deviation was calculated by M change = M1-M2; SD change = (From Cochrane Handbook for Systematic Reviews of Interventions (Version 5.1.0) 16.1.3.2 “Imputing standard deviations for changes from baseline”). To compare between the degrees of carotid stenosis shown on CASUS and those shown on MRA or CTA for the same patient, stenosis shown on MRA or CTA was also quantified using the same CASUS criteria. In view of the fact that the ECST-based classification criteria for vascular stenosis are converted from categorical variables to continuous variables, cubic model curves were used to show the relationship between SS/TSS or RI-TSS and BF or GBF, respectively. ROC curves were used to analyze and compare TSS, RI-TSS, and GBF, with respect to various prognostic indicators (all-cause death, cardiovascular death, recurrent stroke, and composite cardiovascular events), and to calculate the AUC. According to the recommendations by Swets in 1988, [11] an AUC of ≥ 0.7 was considered indicative of an accurate model. *P* values of < 0.05 were considered statistically significant.

## Results

### Patient enrollment and follow-up

During the study period, we treated a total of 968 patients with carotid stenosis, including 107 carotid stent, 15 carotid endarterectomy, and the rest 846 of patients. Of these 846 cases, 96 were excluded according to the inclusion/exclusion criteria (9 with severely impaired consciousness, 20 with mRS scores of > 1, 16 with severe mental disorders or dementia, 21 with serious systemic diseases and with a life expectancy of < 6 months, 8 with a previous history of carotid revascularization, 16 with cardiogenic and cryptogenic stroke, and 6 with critical cerebral infarction). The remaining 750 cases were followed up and observed according to the study design, and the end points were recorded. Among the 750 patients, 58 (7.7%) had recurrent stroke, 26 (3.4%) had non-fatal myocardial infarction, 39 (5.2%) died (all-cause, including 16 cardiovascular death), and 18 (2.4%) were lost to follow-up (Table 2).

### General information

Among the 750 patients, 457 (61%) were male and 293 (39%) were female. The mean age was (64.8 ± 12.4 years).The detailed patient characteristics based on the GBF level quintiles are described in the supplementary Table І. In this group, there were 1500 internal carotid and vertebral arteries, respectively. The total number of carotid arteries with stenosis in whole group was 2602 (86.7%), of which 1311 (87.4%) were ICA, and 1291 (86.1%) were VA.

### Confirmation of the calculated SS for carotid stenosis of varying degrees

After the descriptive statistical analysis, the calculated SS values for ICA with moderate stenosis, severe stenosis, and occlusion were 1.7, 2.8, and 4, respectively. The calculated SS values for VA with moderate stenosis, severe stenosis, and occlusion were 1.6, 2.7, and 4, respectively. The approximate SS values of both ICA and VA with moderate stenosis, severe stenosis, and occlusion were 2, 3, and 4 points, respectively (Table 1).

**Table 1:** Carotid artery blood flow and stenosis score calculation chart for stenosis of varying degrees

Blood vessel	Index	No-stenosis BF	Mild-stenosis BF reduction	Moderate-stenosis BF reduction	Severe-stenosis BF reduction
ICA	BF, ml/Min	418.4 ± 73.5	109.1 ± 76.3	189.4 ± 66.9	301.0 ± 63.8
	Calculated SS, point	0	1	1.7	2.8
	Approximate SS, point	0	1	2	3
VA	BF, ml/Min	172.2 ± 32.9	53.2 ± 31.9	86.5 ± 30.7	141.3 ± 28.5
	Calculated SS, point	0	1	1.6	2.7
	Approximate SS, point	0	1	2	3

*ICA*, internal carotid artery; *VA*, vertebral artery; *BF*, blood flow; *SS*, stenosis score

### The relationship between stenosis and blood flow

A linear relationship was observed between SS and BF in both the ICA and VA (*R*^2^ = 0.734 and 0.783, respectively; both *P* < 0.05) (Supp. Fig. IIA, IIB).

**Fig. 1:**
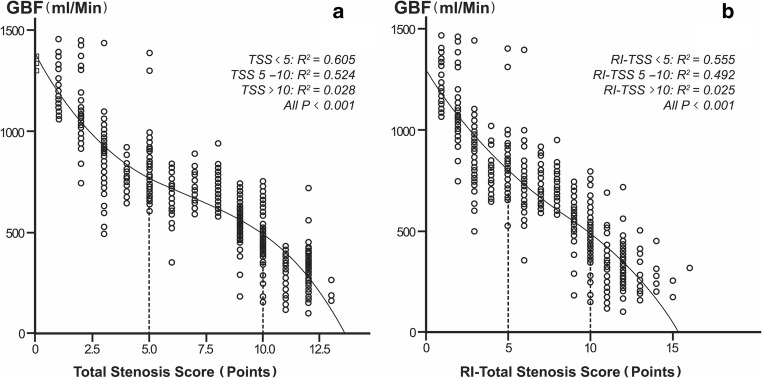
The cubic simulation curve between TSS and GBF (**a**), and between RI-TSS and GBF (**b**)

The relationship between TSS and GBF, and between RI-TSS and GBF could be depicted with inverse “S-shaped” curves as shown in Fig. 1a, b. The fitted equation for the former was *Y* = 1380.6 − 13.3*X* + 24.2*X*2 − 1.2*X*3 (*R*^2^ = 0.839, *P* = 0.006), and that for the latter was *Y* = 1298.2 − 137.4*X* + 9.5*X* − 0.4*X*3 (*R*^2^ = 0.843, *P* = 0.004), where *Y* is GBF and *X* is the TSS or RI-TSS.

Both TSS and RI-TSS were fitted in three segments with threshold values of 5 and 10 (i.e., TSSs of < 5, 5–10, and > 10). When TSS or RI-TSS was 5, the regression model had the best fitting (TSS = 5, *R*^2^ = 0.847, *P* < 0.001; RI-TSS = 5, *R*^2^ = 0.851, *P* < 0.001). With a TSS or RI-TSS of < 5, GBF showed a sharp decline trend after the first peak. With 5 ≤ TSS/RI-TSS ≤ 10, the sharp decline trend of GBF became gradual. With a TSS or RI-TSS of > 10 points, GBF once again showed a sharp decline.

### Analysis of the predictive values of TSS, RI-TSS, and GBF for each end point

**Fig. 2:**
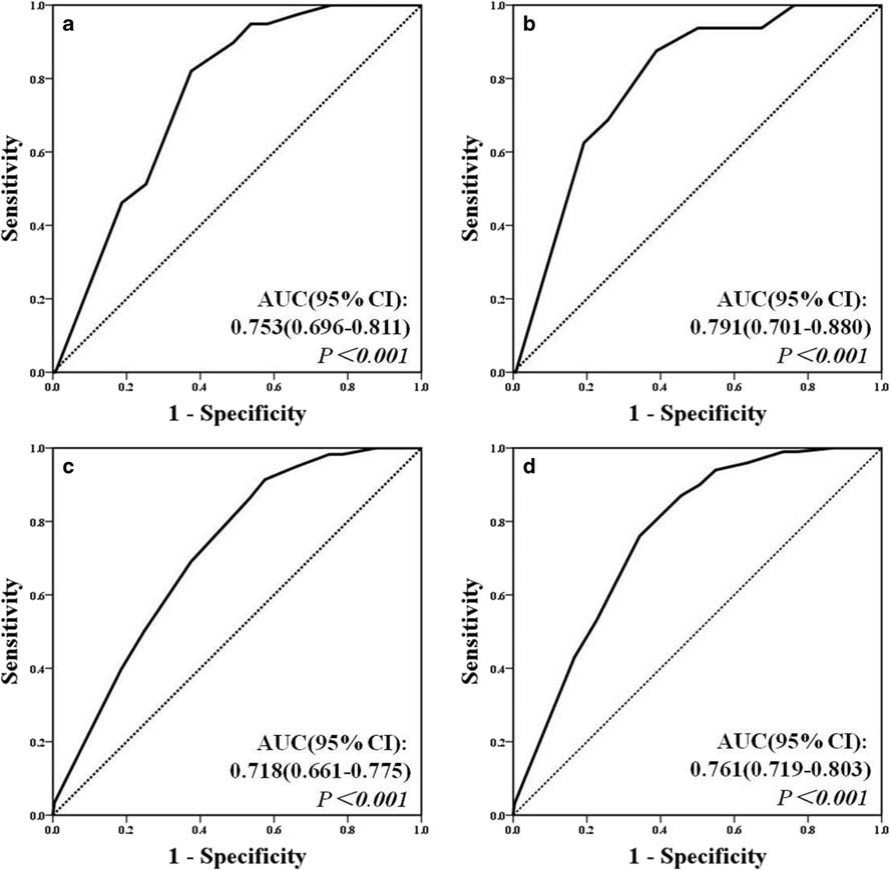
The ROC curves of TSS for predicting clinical outcome in ischemic stroke. **a** All-cause death. **b** Cardiovascular death. **c** Recurrent stroke. **d** Complex cardiovascular events

The ROC analysis revealed that the AUC for all end points predicted by TSS, RI-TSS, and GBF were all > 0.7, and the results were statistically significant (all *P* < 0.001). When comparing the AUC among TSS, RI-TSS, and GBF, no statistically significant differences were found (all *P* > 0.05) (Table 2 and Fig. 2; Supp. Fig. V,VI).

**Table 2:** Comparison of the predictive value of TSS, RI-TSS, and GBF for various endpoint events in ischemic stroke

Endpoint events	Indexes	AUC	SE	95% CI	BPP	P values
All-cause death	TSS	0.753	0.030	0.696–0.811	10	0.167
	RI-TSS	0.759	0.031	0.697–0.821	10	
	GBF	0.744	0.036	0.676–0.811	–	
Cardiovascular death	TSS	0.791	0.046	0.701–0.880	10	0.126
	RI-TSS	0.781	0.047	0.690–0.873	10	
	GBF	0.799	0.048	0.709–0.889	–	
Recurrent stroke	TSS	0.718	0.029	0.661–0.775	7	< 0.001
	RI-TSS	0.713	0.029	0.657–0.770	8	
	GBF	0.721	0.030	0.662–0.780	–	
Non-fatal myocardial infarction	TSS	0.756	0.031	0.695–0.817	9	0.072
	RI-TSS	0.702	0.026	0.622–0.784	9	
	GBF	0.708	0.042	0.627–0.790	–	
Composite cardiovascular events	TSS	0.761	0.019	0.719–0.803	10	< 0.001
	RI-TSS	0.753	0.021	0.711–0.794	*10*	
	GBF	0.754	0.021	0.710–0.798	–	

*TSS*, total stenosis score; *RI*-*TSS*, radiologic image-total stenosis score; *GBF*, global blood flow; *AUC*, area under the curve; *SE*, standard error; *BPP*, best prediction point

## Discussion

In this study, we first excluded 122 patients who underwent carotid stenting and carotid endarterectomy in case these artificially altered vascular stenosis from affecting the study results [4]. The cubic simulation curve analysis showed that the degrees of ICA and VA stenosis negatively correlated with BF; that is, as the degree of stenosis increases, cerebral BF decreases. To objectively verify the rationality of the CASUS scoring standard, we studied the BF reduction with respect to stenosis scores. By using the approximate algorithm of scoring criteria, the calculated SS values of the ICA with moderate stenosis, severe stenosis, and occlusion were 1.7, 2.8, and 4, respectively, and those of VA were 1.6, 2.7, and 4, respectively. The approximate SS values of both ICA and VA were 2, 3, and 4 for moderate stenosis, severe stenosis, and occlusion, respectively. The results suggest that with the increase in carotid stenosis score, the BF decreases linearly. The CASUS quantification scale therefore completely reflected the negative correlation and declining trend, which validated the novel CASUS scoring standard that we proposed. Shakur et al. showed that with the increase in the severity of ICA stenosis, the ipsilateral BF decreased accordingly (when the stenosis was 50–69%, the BF was 197.2 mL/min, and when the stenosis was 70–99%, the BF rate was 130.3 mL/min) [12]. These results were similar in magnitude to the results of the present study. However, in another study by Li Jiamin et al. [13], in which cervical vascular ultrasonography was applied in 69 patients with cerebral infarction, the BF velocities in mild, moderate, and severe ICA stenosis were 948.66 ± 170.33, 647.58 ± 110.65, and 444.19 ± 98.01 mL/min, respectively. The decrease in BF values were somewhat different from the results of this study and the study of Shakur et al. [12],which may be attributed to the differences in the study population, number of cases included, mean age, and most importantly, the study methods.

More and more studies have confirmed that cerebral vascular stenosis first leads to hypoperfusion in the perfusion territory, while increasing cerebral collateral circulation to compensate for impaired cerebral blood flow, and carotid endarterectomy and carotid stent can significantly improve rCBF in the perfusion territory [14–16]. However, the effects of these local vascular stenosis on GBF and the relationship between degree of carotid stenosis and GBF have not been reported. The study data were further analyzed using cubic simulation curves. The relationship between TSS or RI-TSS and GBF showed an inverse “S-shape.” The mechanism of this inverse “S-shaped” relationship is not yet clear, but the main regulation of cerebral BF can be reasonably explained. In the first mechanism regarding the activation and reconstruction of the collateral circulation of the brain, following the stenosis of major vessels, the original anastomotic side vessels dilate and/or form bypasses, and the newly formed or potential vascular anastomotic branches (anterior and posterior communicating arteries, ophthalmic arteries, etc.) play a role in perfusion compensation [17]. In the second mechanism regarding self-regulation, the cerebral vascular system maintains the BF stability by continuously adjusting the vascular tone according to the fluctuating cerebral perfusion pressure [18].In the third mechanism regarding the metabolism-BF coupling regulation, the metabolic activities of the brain accelerate BF, and the coupling of synapses with metabolites (e.g., H+, K+) can cause vascular expansion [19]. In the fourth mechanism regarding neuromodulation, perivascular nerves, vascular endothelial cells, and astrocytes constitute the neurovascular units and are divided into endogenous and exogenous vascular peripheral nerves according to their different origins and neurotransmitters. Exogenous nerves maintain a constant cerebral BF by regulating the vascular tone through the sphenopalatine, supracondylar, and trigeminal ganglia, by regulating cerebral microvasculature through the astrocytes [20]. On the basis of the abovementioned mechanisms of cerebral perfusion regulation, we inferred that when TSS increases initially, the abovementioned regulatory mechanisms have not been fully initiated yet, therefore triggering the initial “sharp decline” characteristics of BF. With the gradual increase in TSS (the gradual increase of carotid stenosis), the collateral cranial circulation in the corresponding site opens up, partially or completely compensating for the BF reduction due to stenosis. For example, if the ICA is severely stenotic, the primary collateral circulation opens, and the posterior and posterior communicating arteries provide BF compensation to the stenotic side. The dynamic balance of cerebral perfusion is therefore reflected as the “plateau” phase of the inverse “S-shaped” curve. When the TSS continues to increase and exceeds the abovementioned cerebral perfusion compensatory potential, a new “sharp decline” appears as the whole brain perfusion volume decreases. We hypothesized that the severely impaired cerebral blood circulation in this phase could lead to the occurrence of an ischemic stroke.

The current methods of assessing carotid stenosis include DSA, CTA, and MRA, of which DSA is regarded as the gold standard diagnostic method [8]. However, it is an invasive test and requires a contrast agent injection. It is expensive and involves risks such as allergic reactions to contrast agents, catheterization-caused vessel wall damage, and embolisms due to the dislocation of unstable plaques. Therefore, DSA cannot be used as a routine examination or primary screening method for carotid stenosis. CTA is valuable as a reference for understanding the nature of carotid artery diseases, the degree of stenosis or occlusion, and the anatomical relationship between stenotic vessels and peripheral vessels and tissues. However, CTA also needs contrast agent injections and hence poses a risk of allergy. MRA does not require injection of contrast agents, and the fine structure of blood vessels can be visualized in high spatial resolution. However, it is expensive and is contraindicated for patients with metallic implants or claustrophobia [21, 22]. In comparison, ultrasonography as a noninvasive imaging test has a specificity of 91.7% and sensitivity of 94.4% for carotid stenosis. It can accurately determine the degree of carotid artery stenosis and is easy to operate, inexpensive, reproducible, safe, and intuitive. Therefore, it is still the test of choice to diagnose and screen carotid artery diseases [23, 24]. Our results showed that the AUC predicted by the TSS values calculated on ultrasonography were > 0.7 for all prognostic end points, and no significant differences in AUC was found between ultrasonographic prediction and CTA, MRA, or GBF (all *P* > 0.05). These results indicate that TSS measured using carotid artery CDUS has a good predictive value for the occurrence of cardiovascular and cerebrovascular diseases. Because the difference in sensitivity and specificity between TSS and IR-TSS in not significant, TSS is no less than that of CTA and MRA in predicting cardiovascular and cerebrovascular disease recurrence.

This study has its limitations. First, the study population is from a single institution, which could be a certain selection bias. Second, this study did not dynamically monitor the long-term intracranial BF changes and therefore cannot directly reveal the effect of the compensatory collateral circulation and cerebral perfusion adjustments caused by intracranial and external chronic stenosis or occlusion on CASUS. In addition, the degree and location of stenosis showed interpatient variations. In most cases, the major actor may only be the responsible artery or the segment with the most severe stenosis, but this study used total stenosis to predict clinical prognosis, and the overall predictive value of the CASUS standard has been confirmed. Third, the prognosis of stroke is affected by various factors such as the burden of carotid atherosclerosis, carotid plaque property, and carotid intima-media thickness. In addition, a small number of patients in our study had received intravenous thrombolytic therapy. Therefore, this study was limited because it investigated the CASUS criteria for predicting clinical outcomes just standing on the shore of total carotid stenosis. Being the first study to explore the novel CASUS standard, a system somewhat different from the conventional criteria for evaluating carotid stenosis, the efficacy and reliability of the method still need to be confirmed by future large-sample, long-term, and multi-center comparative study.

## Conclusions

Taken together, the results of this study suggest that the carotid artery stenosis ultrasound scale can better reflect the level of cerebral reperfusion and can better predict the occurrence of cardiovascular and cerebrovascular diseases.

## Electronic supplementary material

ESM 1 (DOCX 940 kb)
