# Supplementary material for: The role of carotid stenosis ultrasound scale in the prediction of ischemic stroke
Source: Neurol Sci. 2020 Jan 3;41(5):1193–9. doi: 10.1007/s10072-019-04204-8 (PMC7196942; doi:10.1007/s10072-019-04204-8)
Supplement: Supplementary file 1 — (DOCX 940 kb) [file 10072_2019_4204_MOESM1_ESM.docx]

**SUPPLEMENTAL MATERIAL**

**The Role of carotid stenosis ultrasound scale in the prediction of ischemic stroke**

**Contents**

Supplemental methods ………………………………………………………………………3

Study protocols ………………………………………………………………………………3

Stenosis degree and blood flow assessments…………………………………………………3

Data definitions ………………………………………………………………………………5

Angiographic assessments……………………………………………………………………6

Treatments……………………………………………………………………………………8

Supplemental Figures ………………………………………………………………………9

FigureⅠ Patient flow and stenosis statistics ………………………………………………10

FigureⅡ The cubic simulation curve between SS and BF for the ICA extra-cranial segment and the VA V1—V3 segment………………………………………………………………11

Figure Ⅲ Ultrasound image comparison of ICA stenosis of varying degrees………………12

Figure Ⅳ Ultrasound image comparison of VA stenosis of varying degrees………………13

Figure Ⅴ The ROC curves of RI-TSS for predicting clinical outcome in ischemic stroke…14

Figure Ⅵ The ROC curves of GBF for predicting clinical outcome in ischemic stroke …15

Supplemental Tables…………………………………………………………………………15

Supplemental References……………………………………………………………………16

**Supplemental methods**

**Study protocols**

This study was a single-institution, prospective cohort study. The risk assessment indicators included in this study were based on the content of the “Stroke Recurrence Risk Assessment Form” published by the “Stroke Risk Factor Screening and Intervention” project of Chinese National Institute of Health and expanded as appropriate. The study was approved by the Ethics Committee of the Lianyungang Hospital Affiliated to Xuzhou Medical University, and written informed consent was obtained from each patient or his/her proxy.

**Stenosis degree and** **blood flow assessments**

All patients included in the study completed at least one carotid ultrasonographic examination within 7 days of stroke onset. The degree of carotid artery stenosis was assessed by an attending physician specialized in ultrasonography imaging who was blinded to the follow-up data. A color Doppler ultrasonography (CDUS) system, Vivid E7 (GE, USA), was used for the image acquisition. The patient was placed in a supine position, with the neck neutrally extended and the head slightly turned to the contralateral side. By using a 9-L line array probe to perform a full-scale continuous scan from the bottom to the top, starting from the root of the common carotid artery and continuing along the carotid arteries, sagittal and axial scans were acquired. Two-dimensional gray-scale images were first used to observe the arterial wall, intraluminal structure, and echo. Color Doppler imaging was then used to observe the BF, finally followed by the collection of the hemodynamic parameters of the bilateral common carotid arteries, extracranial segment of the ICA, and V1–V3 segment of the VA using pulse Doppler imaging. For those with a relatively short and stout neck and a relatively superior bifurcation, the C4-8L microprotrusion array probe was used in combination, especially at the opening of the VA. Owing to the varying duration at the carotid lumen stenosis distal to a stenosis, negative reconstruction changes are often observed. To minimize their impact on the accuracy of the stenosis rate calculation, the study adopted the ECST method 13 to calculate the stenosis rate. The diameter stenosis rate was calculated using the measured diameter of the arterial lumen at the narrowest point (A) and the diameter of the original arterial lumen (B) as the diameter stenosis rate (%) = (B − A)/B × 100%. Other hemodynamic parameters were also used in combination to evaluate the stenosis, including peak systolic velocity (PSV), end-diastolic velocity (EDV), time-averaged mean velocity (TAMV), and the ratio of BF velocity at stenosis to that at a normal segment distal to the stenosis. According to Chinese stroke vascular ultrasound guidance specification by the 2015 National Health and Family Planning Commission Stroke Prevention and Control Commission[15] ,the carotid artery stenosis was divided into four levels as follows: For the ICA, mild stenosis of <50% (PSV < 125 cm/s and EDV < 40 cm/s), moderate stenosis of 50%–69% (125 ≤ PSV < 230 cm/s and 40 ≤ EDV < 100 cm/s), severe stenosis of 70%–99% (PSV ≥ 230 cm/s and EDV ≥ 100 cm/s), and complete occlusion (no flow signal; see the supplementary Fig.Ⅰ). For the VA, mild stenosis of <50% (85 < PSV < 140 cm/s and 27 < EDV < 35 cm/s), moderate stenosis of 50%–69% (140 ≤ PSV < 220 cm/s and 35 ≤ EDV < 50 cm/s), severe stenosis of 70%–99% (PSV ≥ 220 cm/s and EDV ≥ 50 cm/s), and complete occlusion (no flow signal;see the supplementary Fig.Ⅱ).

Blood flow (the amount of blood flow through a given cross-section of a blood vessel per unit time, i.e., the product of the linear velocity of the BF and the cross-sectional area of the blood vessel lumen) was measured using the automatic detection function of the CDUS system at the stenosis and in the proximal and distal segments with respect to the stenosis. The average of the three measurements was used to represent the BF of the vessel with stenosis. To improve the reliability and accuracy of the measurement, data were measured for 5 consecutive cardiac cycles, and the stenosis rate and BF (mL/min) calculated for each cycle were averaged. Cerebral BF was calculated using the following formula[16]:1) cross-sectional area (S) = (A/2)2 × π; 2) BF volume per unit area = TAMV × S × 60; 3) total VA BF = left VA BF + right VA BF; 4) total ICA BF = left ICA BF + right ICA BF; and 5) GBF = total VA BF + total ICA BF.

**Data definitions**

In accordance with definition of stroke published in 2013 by the AHA/ASA ,^1^ a case was considered as acute ischemic stroke if it met the following 5 conditions: 1. acute onset; 2. focal neurological function deficit (a small number may have a comprehensive neurological deficit); 3. no requirement on the duration of symptoms or signs if imaging shows responsible lesions, otherwise the symptoms or signs need to last >24 hours; 4. cerebral hemorrhage has been ruled out by CT/MRI; and 5. non-vascular causes have been ruled out. The critical cerebral infarction was defined as as the dying cerebral infarction patient in continuous rescue. The onset time of the stroke was defined as the last time with no neurological impairment to the patient’s knowledge; or for patients who woke up with stroke symptoms, the onset time of the stroke was considered to be the time when the patient fell asleep. The V1-V3 segment of the VA was defined as from the beginning of the VA to the occipital foramen.^2^ Symptomatic carotid stenosis was defined as carotid stenosis with one or more of the following symptoms: transient amaurosis, transient ischemic attack (TIA), mild or non-disabling stroke caused by craniocerebral vasculopathy within the past six months .^3^ Atrial fibrillation was diagnosed by the electrocardiographic (EKG) findings at the time of onset. Coronary heart diseases were defined as coronary artery atherosclerosis causing stenosis or obstruction of the lumen, resulting in heart conditions manifested as myocardial ischemia, hypoxia, or necrosis. Heart failure was defined as a group of clinical syndromes due to structural or functional abnormalities of the heart leading to impaired ventricular filling or ejection, usually manifested as dyspnea, impaired activity level, as well as fluid retention (pulmonary congestion and peripheral edema). Chronic kidney diseases were defined as structural or functional renal abnormalities >3 months.^4^ In accordance with the European arterial hypertension management practice guidelines published in 2013,^5^ hypertension was defined as SBP ≥ 140 mmHg and/or DBP ≥ 90 mmHg in three measurements on different days without any hypertension medication, or anyone with a hypertension history and currently on hypertension medications (including those whose BP <140/90 mmHg). Diabetes was defined according to the ADA Diabetes Medical Treatment Standards published in 2015. ^6^ The body mass index (BMI) = weight/height^2^ (kg/m^2^) was calculated with the height and weight of the patient as measured at the time of admission.

**Angiographic assessments**

The CTA scans used the Somatom Definition Flash CT system (Siemens Medical Systems, Germany). The patient was placed supine and scanned from the aortic arch to the top of the head. Contrast agent tracking was used in selected regions of interest at the aortic arch or carotid arteries to monitor the CT numbers. When the CT number exceeded 100 Hounsfield Units, the scan was automatically triggered with a delay of 4 seconds. Scanning parameters were as follows: gantry rotation time = 0.33 s, scan time = 8-12 s, tube voltage = 120 kVp, and tube current = 110 mA. A double-barreled syringe was used to inject 40.0 mL of non-ionic contrast agent iohexol (containing 370 mg/mL iodine) and 20.0 mL of saline intravenously from the cubital vein. The injection flow rate was 4.5 mL/s. The raw data were post-processed and reconstructed on a Flash DSCT workstation Syngo (Siemens Medical Systems, Germany). Advanced reconstructions such as surface reconstruction, maximum intensity projection (MIP), multi-planar reconstruction (MPR), and volume reconstruction (VR) were obtained and displayed on the workstation.

A 3.0T superconducting Signa MRI system (GE, USA) was used for the MRA examination with a 16-channel head orthogonal coil or head-neck coil. The patient was supine and the scan was from the aortic arch to the top of the head. Scanning parameters were as follows: T1WI axial with TR=2630 ms and TE=25 ms; FSE T2WI axial with TR=4600 ms and TE=110 ms; FLAIR with TR=9600 ms, and TE=115 ms; DWI with TR=6200 ms and TE=80 ms,and b value = 0 and 1000/mm^2,^ respectively; 3D-TOF MRA with TR=20 ms and TE=3.0 ms; FOV=24 cm×24 cm, slice thickness=5.0 mm, and slice spacing=1.5 mm. The raw data were processed on an ADW 4.3 workstation (GE, USA) with the Functool software package. The MIP image was calculated using the acquired MRA data.

**Treatments**

In accordance with the AHA/ASA guidelines for the early management of patients with acute ischemic stroke ,^7^ basic treatments were given to the patients according to their conditions, such as giving medications for platelet anti-aggregation, lipid regulation, anticoagulation, intracranial pressure reduction, blood pressure control, and blood glucose control. After discharge, the patients strictly followed the AHA/ASA guidelines for the prevention of stroke in patients with stroke and transient ischemic attack^8^ to receive measures of secondary prevention such as anti-aggregation, anticoagulation, lipid regulation, blood pressure control, blood glucose control, and rehabilitation.

**Supplemental Figures**


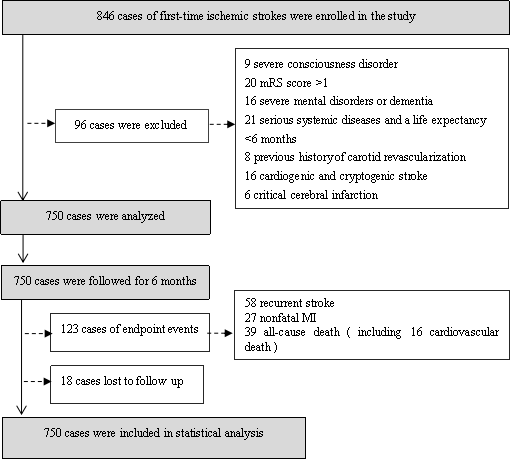


**Figure****Ⅰ Patient flow and stenosis statistics (ICA, internal carotid artery; VA, vertebral artery)**

**
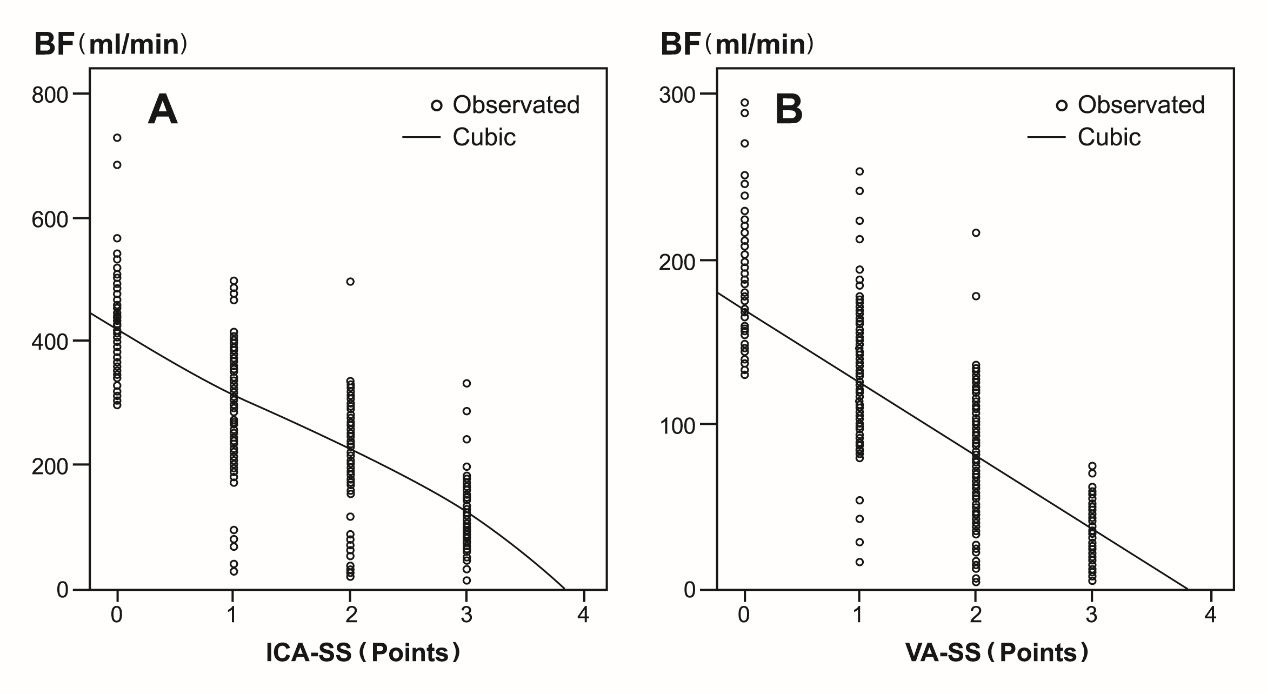
**

**Figure Ⅱ The cubic simulation curve between SS and BF for the ICA extra-cranial segment ( A ) and the VA V1—V3 segment ( B )**


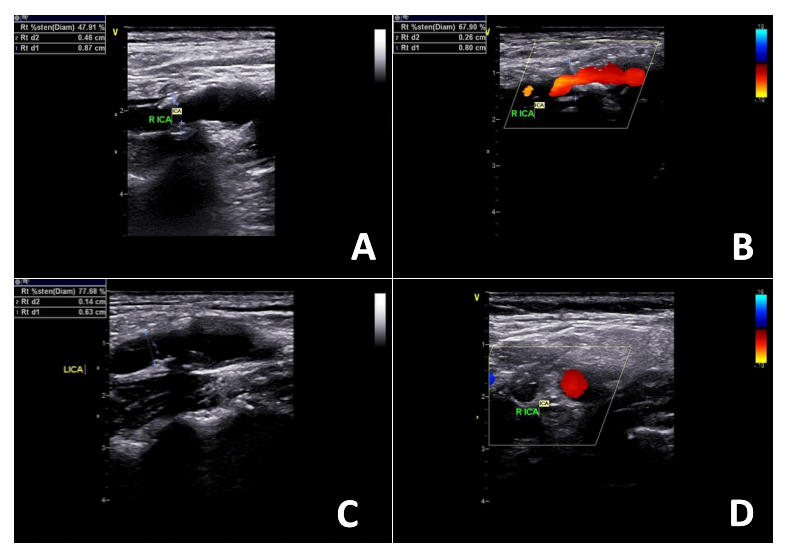


**Figure Ⅲ Ultrasound image comparison of ICA stenosis of varying degrees** A, mild stenosis; B, moderate stenosis; C, severe stenosis; D, occlusion.


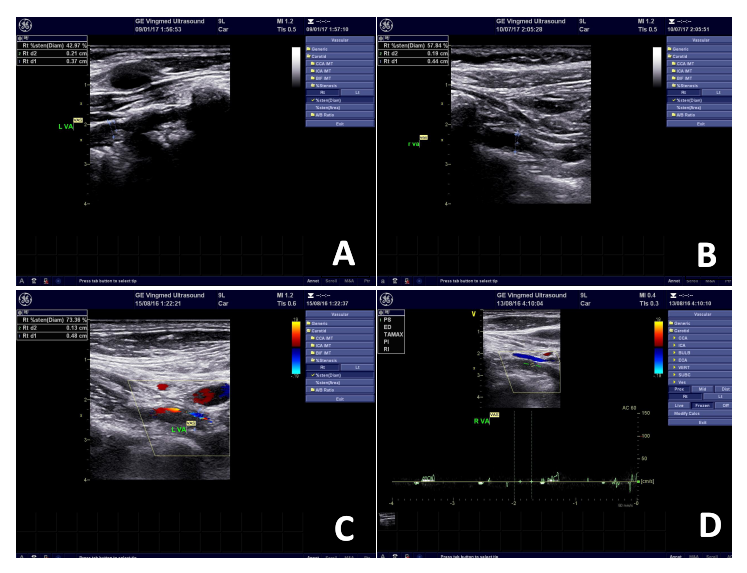


**Figure Ⅳ Ultrasound image comparison of VA stenosis of varying degrees** A, mild stenosis; B, moderate stenosis; C, severe stenosis; D, occlusion.

**
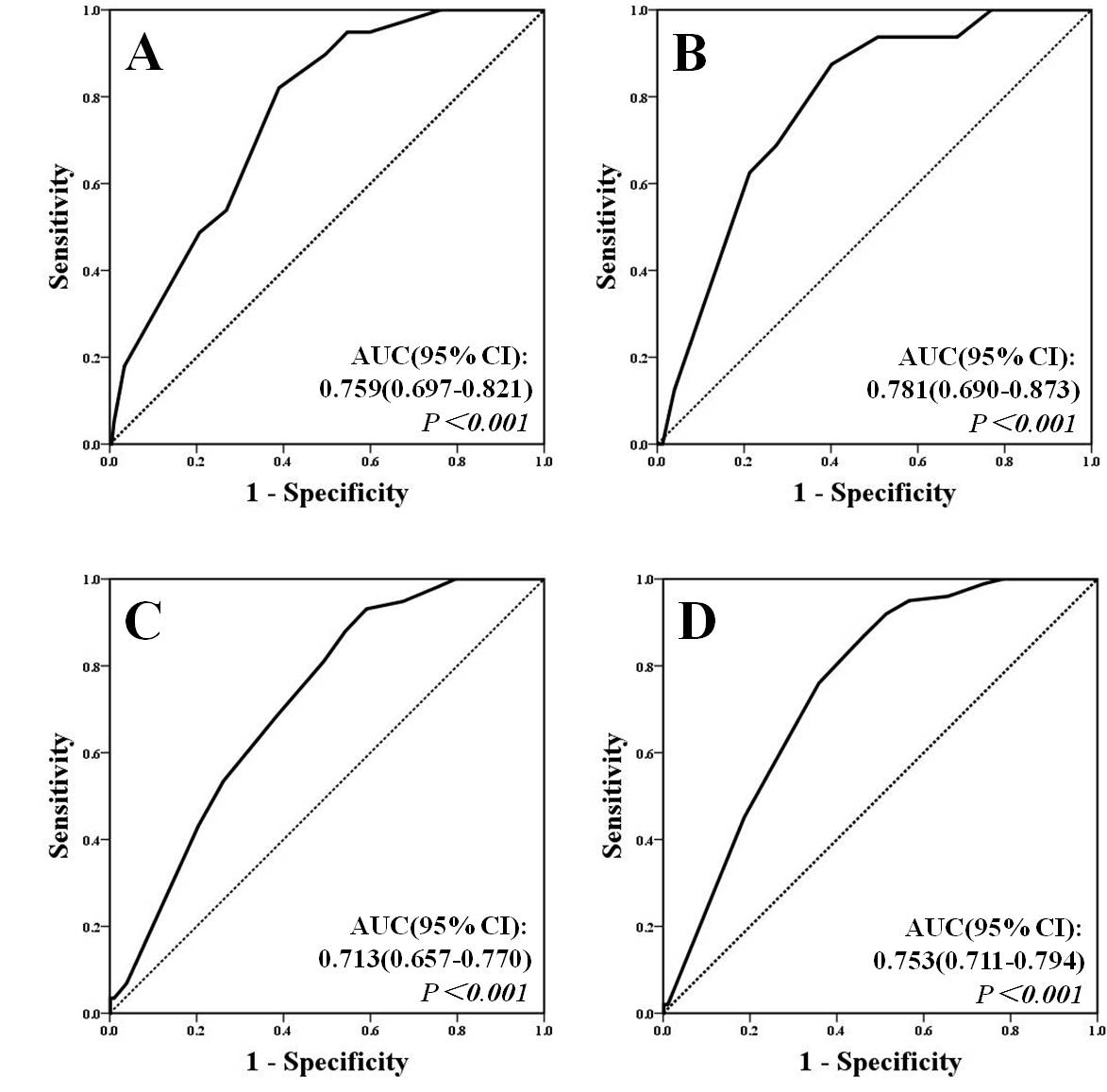
**

**Figure Ⅴ The ROC curves of RI-TSS for predicting clinical outcome in ischemic stroke** A, all-cause death; B, cardiovascular death; C, recurrent stroke; D, complex cardiovascular events.

**
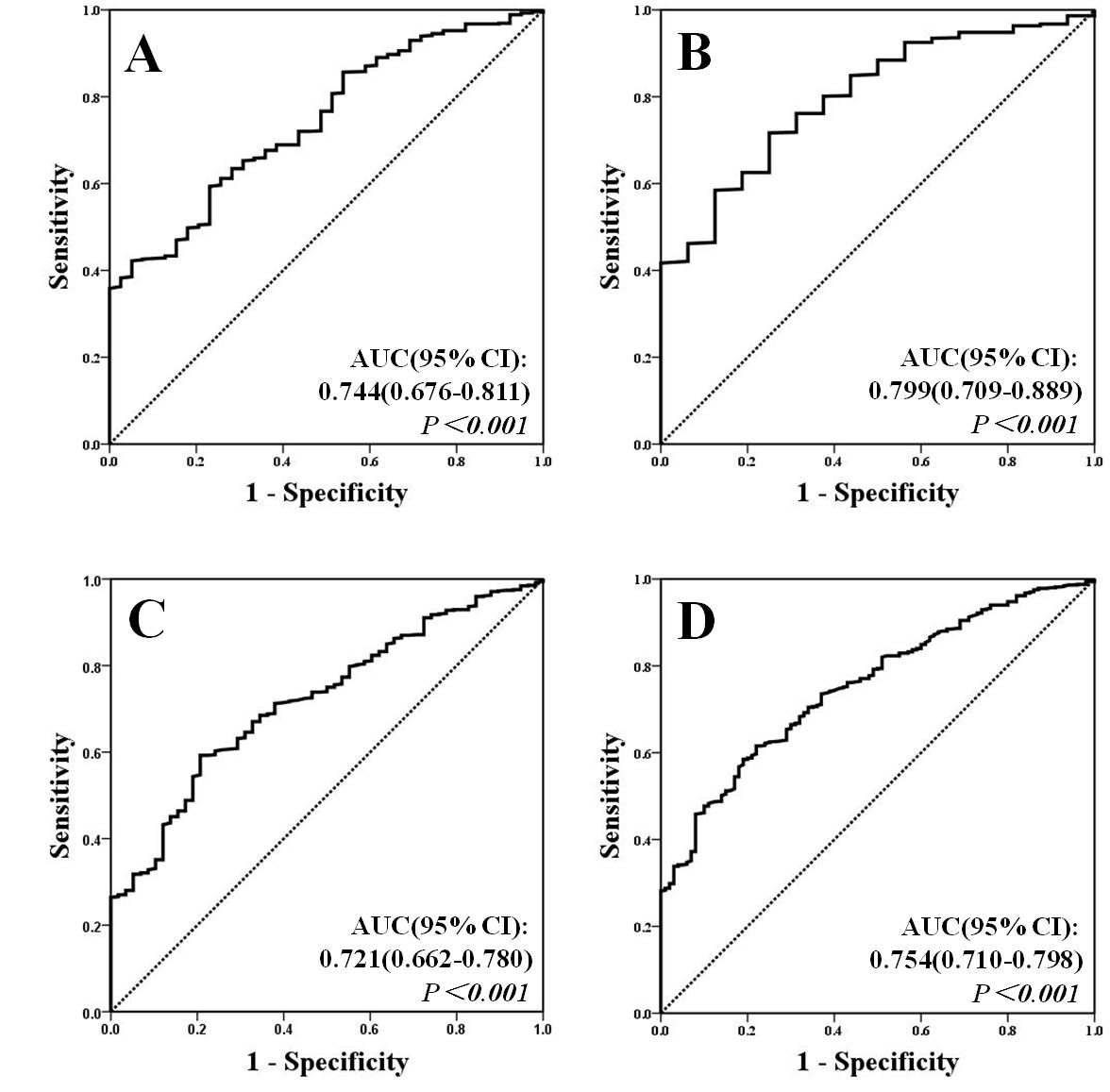
**

**Figure Ⅵ The ROC curves of GBF for predicting clinical outcome in ischemic stroke** A, all-cause death; B, cardiovascular death; C, recurrent stroke; D, complex cardiovascular events.

**Supplemental Table**

**Table I The characteristics of patients based on the GBF level of quintile groups**

| **Background characteristics** | **Q1**  **(<337)** | **Q2**  **( 337-526)** | **Q3**  **( 527-687)** | **Q4**  **( 688-850)** | **Q5**  **(＞850)** | ***P value*** |
| --- | --- | --- | --- | --- | --- | --- |
| **General characteristics** |  |  |  |  |  |  |
| Age（year） | 65.4±12.4 | 65.1±12.6 | 64.5±12.4 | 64.4±13.3 | 64.6±11.7 | *0.769* |
| Female, n（%） | 52(17.7) | 63(21.5) | 53(18.1) | 62(21.2) | 63(21.5) | *0.508* |
| BMI（kg/m^2^） | 24.7±3.1 | 24.9±3.2 | 25.5±3.1 | 25.0±3.6 | 24.9±3.5 | *0.265* |
| Current smoker, n（%） | 70(23.0) | 56(18.4) | 65(21.4) | 52(17.1) | 61(20.1) | *0.066* |
| SBP（mmHg） | 146.1±21.1 | 145.7±19.2 | 148.8±21.7 | 147.8±19.1 | 150.0±23.0 | *0.335* |
| DBP（mmHg） | 85.4±12.1 | 86.0±11.5 | 86.6±12.6 | 86.6±11.8 | 87.4±13.4 | *0.676* |
| NIHSS（point） | 9.3±2.7 | 9.5±2.9 | 8.7±2.2 | 7.3±2.6 | 6.8±1.9 | *0.002* |
| mRS（point） | 3.0±1.2 | 2.7±1.2 | 2.5±1.2 | 2.5±1.2 | 2.5±1.2 | *0.000* |
| HR（beat/minute） | 74.5±7.3 | 74.4±7.4 | 74.9±9.0 | 74.9±10.9 | 76.1±11.5 | *0.504* |
| **Medical history** |  |  |  |  |  |  |
| Hypertension，n（%） | 96(18.9) | 105(20.7) | 105(20.7) | 100(19.7) | 101(19.9) | *0.885* |
| Diabetes，n（%） | 35(19.6) | 44(24.6) | 40(22.3) | 35(19.6) | 25(14) | *0.134* |
| Coronary heart ，n（%） | 19(26.8) | 14(19.7) | 11(15.5) | 16(22.5) | 11(15.5) | *0.435* |
| Heart failure，n（%） | 2(20.0) | 2(20.0) | 1(10.0) | 2(20.0) | 3(30.0) | *0.656* |
| Atrial fibrillation，n（%） | 13(24.1) | 11(20.4) | 9(16.7) | 11(20.4) | 10(18.5) | *0.916* |
| Chronic kidney disease，n（%） | 0(0.0) | 0(0.0) | 1(20.0) | 2(40.0) | 2(40.0) | *0.057* |
| **Inpatient treatment** |  |  |  |  |  |  |
| Thrombolysis，n（%） | 3(27.3) | 4(36.4) | 1(9.1) | 2(18.2) | 1(9.1) | *0.194* |
| Glucose-lowering，n（%） | 35(21.5) | 35(21.5) | 41(25.2) | 30(18.4) | 22(13.5) | *0.104* |
| Lipid-lowering，n（%） | 147(19.8) | 149(20.1) | 150(20.2) | 149(20.1) | 148(19.9) | *0.996* |
| Anti-platelet aggregation，n（%） | 145(19.8) | 147(20.1) | 148(20.2) | 150(20.5) | 143(19.5) | *0.607* |
| Anticoagulation，n（%） | 11(22.4) | 12(24.5) | 8(16.3) | 10(20.4) | 8(16.3) | *0.884* |
| Blood pressure-lowering，n（%） | 42(14.6) | 59(20.5) | 60(20.8) | 64(22.2) | 63(21.9) | *0.078* |

BMI, Body Mass Index; SBP, Systolic Blood Pressure; DBP, Diastolic Blood Pressure; NIHSS, The National Institutes of Health Stroke Scale; mRS, modified Rankin Scale; HR, Heart Rate.

**Supplemental References**

1. [Sacco RL](https://www.ncbi.nlm.nih.gov/m/pubmed/?term=Sacco%20RL%5bAuthor%5d&sort=ac&from=/23652265/ac), [Kasner SE](https://www.ncbi.nlm.nih.gov/m/pubmed/?term=Kasner SE[Author]&sort=ac&from=/23652265/ac), [Broderick JP](https://www.ncbi.nlm.nih.gov/m/pubmed/?term=Broderick%20JP%5bAuthor%5d&sort=ac&from=/23652265/ac), [Caplan LR](https://www.ncbi.nlm.nih.gov/m/pubmed/?term=Caplan%20LR%5bAuthor%5d&sort=ac&from=/23652265/ac), [Connors JJ](https://www.ncbi.nlm.nih.gov/m/pubmed/?term=Connors%20JJ%5bAuthor%5d&sort=ac&from=/23652265/ac), [Culebras A](https://www.ncbi.nlm.nih.gov/m/pubmed/?term=Culebras A[Author]&sort=ac&from=/23652265/ac), et al. An updated definition of stroke for the 21st century: a statement for healthcare professionals from the American Heart Association/American Stroke Association. *Stroke* 2013, **44**(7):2064-2089.
2. National Health and Family Planning Commission Stroke Prevention Project Committee. Guidelines on blood vessel ultrasound tests for stroke. *Chinese Journal of Medical Ultrasound* 2015,**12**(8):599-610．
3. Guo Wei, Fu Weiguo, Chen Zhong’s translation. Rutherford's Vascular Surgery. Seventh Edition, Beijing: Peking University Medical Press, 2013.
4. Kdigo. Kdigo 2012 Clinical Practice Guideline for the Evaluation and Management of Chronic Kidney Disease. *Kidney Int Suppl* 2013, **3**(1): 1-150.
5. [Mancia G](https://www.ncbi.nlm.nih.gov/m/pubmed/?term=Mancia%20G%5bAuthor%5d&sort=ac&from=/24359485/ac), [Fagard R](https://www.ncbi.nlm.nih.gov/m/pubmed/?term=Fagard R[Author]&sort=ac&from=/24359485/ac), [Narkiewicz K](https://www.ncbi.nlm.nih.gov/m/pubmed/?term=Narkiewicz K[Author]&sort=ac&from=/24359485/ac), [Redon J](https://www.ncbi.nlm.nih.gov/m/pubmed/?term=Redon%20J%5bAuthor%5d&sort=ac&from=/24359485/ac), [Zanchetti A](https://www.ncbi.nlm.nih.gov/m/pubmed/?term=Zanchetti A[Author]&sort=ac&from=/24359485/ac), [Böhm M](https://www.ncbi.nlm.nih.gov/m/pubmed/?term=B%C3%B6hm M[Author]&sort=ac&from=/24359485/ac), et al.2013 ESH/ESC practice guidelines for the management of arterial hypertension: the task force for the management of arterial hypertension of the European Society of Hypertension (ESH) and of the European Society of Cardiology (ESC).*Eur Heart J* 2013, **34**(28):2159.
6. [American Diabetes Association](http://guide.medlive.cn/publisher/54).Standards of Medical Care in Diabetesd 2015. *Diabetes Care* 2015, **38** (Supplement 1):S1-S93.
7. [Jauch EC](https://www.ncbi.nlm.nih.gov/m/pubmed/?term=Jauch%20EC%5bAuthor%5d&sort=ac&from=/23370205/ac), [Saver JL](https://www.ncbi.nlm.nih.gov/m/pubmed/?term=Saver%20JL%5bAuthor%5d&sort=ac&from=/23370205/ac), [Adams HP Jr](https://www.ncbi.nlm.nih.gov/m/pubmed/?term=Adams%20HP%20Jr%5bAuthor%5d&sort=ac&from=/23370205/ac), [Bruno A](https://www.ncbi.nlm.nih.gov/m/pubmed/?term=Bruno%20A%5bAuthor%5d&sort=ac&from=/23370205/ac), [Connors JJ](https://www.ncbi.nlm.nih.gov/m/pubmed/?term=Connors%20JJ%5bAuthor%5d&sort=ac&from=/23370205/ac), [Demaerschalk BM](https://www.ncbi.nlm.nih.gov/m/pubmed/?term=Demaerschalk BM[Author]&sort=ac&from=/23370205/ac), et al. Guidelines for the early management of patients with acute ischemic stroke. *Stroke* 2013, **44**(3):870-947.
8. [Kernan WN](https://www.ncbi.nlm.nih.gov/m/pubmed/?term=Kernan%20WN%5bAuthor%5d&sort=ac&from=/24788967/ac), [Ovbiagele B](https://www.ncbi.nlm.nih.gov/m/pubmed/?term=Ovbiagele B[Author]&sort=ac&from=/24788967/ac), [Black HR](https://www.ncbi.nlm.nih.gov/m/pubmed/?term=Black%20HR%5bAuthor%5d&sort=ac&from=/24788967/ac), [Bravata DM](https://www.ncbi.nlm.nih.gov/m/pubmed/?term=Bravata DM[Author]&sort=ac&from=/24788967/ac), [Chimowitz MI](https://www.ncbi.nlm.nih.gov/m/pubmed/?term=Chimowitz MI[Author]&sort=ac&from=/24788967/ac), [Ezekowitz MD](https://www.ncbi.nlm.nih.gov/m/pubmed/?term=Ezekowitz MD[Author]&sort=ac&from=/24788967/ac), et al. Guidelines for the prevention of stroke in patients with stroke and transient ischemic attack. *Stroke* 2014, **45**(7):2160-236.
